# The accuracy and completeness of drug information in Google snippet blocks

**DOI:** 10.5195/jmla.2021.1229

**Published:** 2021-10-01

**Authors:** Cambrey Nguyen

**Affiliations:** 1 cnguyen@ku.edu, Clinical Assistant Professor, Drug Information, Pharmacy Practice, University of Kansas School of Pharmacy, Lawrence, KS

**Keywords:** Internet, pharmacists, drug information, patient care, search engine

## Abstract

**Introduction:**

Consumers commonly use the Internet for immediate drug information. In 2014, Google introduced the snippet block to programmatically search available websites to answer a question entered into the search engine without the need for the user to enter any websites. This study compared the accuracy and completeness of drug information found in Google snippet blocks to US Food and Drug Administration (FDA) medication guides.

**Methods:**

Ten outpatient drugs were selected from the 2018 Clinical Drugstats Database Medical Expenditure Panel Survey. Six questions in the medication guide for each drug were entered into the Google search engine to find the snippet block. The accuracy and completeness of drug information in the Google snippet block were quantified by two different pharmacists using a scoring system of 1 (less than 25% accurate/complete information) to 5 (100% accurate/complete information). Descriptive statistics were used to summarize the scores.

**Results:**

For five out of the six questions, the information in the Google snippets had less than 50% accuracy and completeness compared to the medication guides. The average accuracy and completeness scores of the Google snippets were highest for “What are the ingredients of [the drug]?” with scores of 3.38 (51–75%) and 3.00 (51–75%), respectively. The question on “How to take [drug]?” had the lowest score with averages of 1.00 (<25%) for both accuracy and completeness.

**Conclusion:**

Google snippets provide inaccurate and incomplete drug information when compared to FDA-approved drug medication guides. This aspect may cause patient harm; therefore, it is imperative for health care and health information professionals to provide reliable drug resources to patients and consumers if written information may be needed.

## INTRODUCTION

A survey conducted by the Pew Research Center for Internet and Technology found that 59% of adults used the Internet to obtain health information in the past year [1], and of those individuals, 80% began their search with Google [2]. With the Google search engine being so accessible to consumers for health-related information, it is important for health care professionals and health information professionals to understand how information is searched online and the types of sources that may provide the information that is found. Moreover, the quality and reliability of drug information found online remains a key concern among health care professionals and health information professionals [3].

In 2014, Google introduced a new feature called the snippet block to assist users with their searches. This feature programmatically searches available websites using an algorithm to answer the question entered into the search bar without requiring the user to enter any websites. The answer to the inquiry obtained by Google is displayed within a box at the top of the results page for the user [4], offering consumers quicker access to health-related information. A news article on National Public Radio (NPR) first highlighted the Google snippet feature in which a consumer asked about eating eggs during pregnancy on the Google search engine [5]. The Google snippet block provided an answer that eggs should be fully cooked to kill the bacteria; however, salmonella poisoning does not pose any fetal risks. As noted in the NPR feature, this answer was only partially correct: raw eggs may pose harm to a fetus if the mother is infected with the bacteria found in the eggs.

A study conducted in 2019 further highlighted the frequency of inaccurate information retrieved through the Google search engine [6]. The study used the top ten health questions asked on Google from 2018, which included “What is a keto diet,” “What is ALS disease,” and “What is endometriosis.” Using these questions, the study assessed the quality of information provided by the featured snippet and knowledge panel, finding that the information retrieved from open-sourced websites (e.g., medicalnewstoday.com) was not reliable. In addition to health information, consumers frequently utilize Google, and its snippet block feature, to search for information about prescription and over-the-counter drugs [7–10].

This study was conducted to compare the accuracy and completeness of drug information found in Google snippet blocks to the US Food and Drug Administration (FDA) medication guides of ten outpatient drugs. The secondary objective was to identify the references cited by the Google snippet blocks.

## METHODS

The list of drugs included in the analysis for this study was obtained from the 2018 Clinical Drugstats Database Medical Expenditure Panel Survey most commonly prescribed outpatient medications. From the list, medications that had a corresponding medication guide available from the FDA website were selected. The medication guides were chosen as the standard of comparison as they are reviewed and regulated by the FDA. In addition, medication guides are created for patients and contain information deemed necessary to prevent patient harm, allow patients to make an informed decision about medication use, and/or provide adherence information essential to product efficacy [11].

All the medication guides were obtained from the FDA website on the same day in December 2019 to avoid discrepancies caused by updates or changes. Within the medication guides, there were six key questions selected to obtain the Google snippet blocks: “What is [drug] used for?”, “How do I take [drug]?”, “What are the possible side effects of [drug]?”, “What should I avoid while taking [drug]?”, “What are the ingredients of [drug]?”, and “How do I store [drug]?”

**Figure 1:**
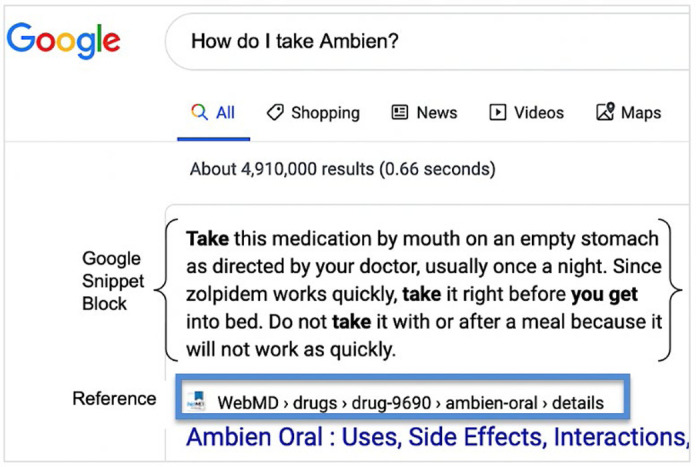
A screenshot of a Google snippet block when a question is entered into the search bar. Google and the Google logo are trademarks of Google LLC, and this article is not endorsed by or affiliated with Google in any way.

Each question for all of the drugs was entered into the Google search engine, and a screen capture of the Google snippet block was taken (Figure 1). If a question did not initially yield a Google snippet block, the question was reworded to produce a Google snippet block without losing the meaning of the original question (e.g., “how should I take [drug]” or “what to avoid while taking [drug]”). All Google snippet blocks for the questions were obtained on the same day in December 2019 as the medication guides.

Accuracy was defined as the amount of factual information, and completeness was defined as the amount of information that overlapped with the medication guide. The accuracy and completeness of drug information in the Google snippets were quantified using a scoring system of 1 (less than 25% accurate/complete information) to 5 (100% accurate/complete information) (Table 1). The scoring system was based on previous studies that evaluated accuracy and/or completeness of drug information, but included more increments to provide more precise ratings of the drug information [7, 8].

**Table 1:** Rating scale to assess accuracy and completeness

Score	Definition
**1**	<25% of information is accurate/complete
**2**	26–50% of information is accurate/complete
**3**	51–75% of information is accurate/complete
**4**	76–99% of information is accurate/complete
**5**	100% of information is accurate/complete

To ensure consistency among the raters using the adapted scoring system, two pharmacists, including the principal investigator, conducted an inter-rater reliability test (IRR) to rate accuracy and completeness of the drug information. Each question may receive a maximum of 5 points in each accuracy and completeness category. For the IRR test, eight drugs (Lexapro, Prozac, Coumadin, Cymbalta, Trillipix, Actos, Cipro, Colcrys) not included in the final analysis were selected and independently scored by each pharmacist. The intraclass correlation coefficient (ICC), two-way mixed effects with absolute agreement, was calculated to be 0.843, which was considered to be excellent agreement.

Ten different drugs were then selected for the final analysis and scored according to the aforementioned process. Discrepancies among the ratings were discussed and resolved by consensus. The source of information cited by the Google snippets for each of the questions and the frequency in which they were cited were also collected.

Scores provided by each pharmacist for the IRR test and the ICC were analyzed using SPSS, v25 (IBM SPSS, Armonk, NY). Descriptive statistics were used to summarize the scores for drugs with data presented as means with standard deviations. IRB oversight was exempted for this study as human participants were not included and human data were not collected.

## RESULTS

A total of fifty-five Google snippet blocks were retrieved from the Google engine, as five questions did not have a Google snippet block available. There were nine questions that did not have the corresponding drug information in the medication guides for comparison; therefore, a total of forty-six Google snippet blocks were available for analysis. There were a total of seven drugs that did not have Google snippet blocks available or information for the question in the medication guide or a combination of the two. The average accuracy and completeness ratings for each drug are provided in Table 2.

**Table 2:** Mean accuracy and completeness scores by drug

Drug	Accuracy mean (SD)	Completeness mean (SD)
Ambien	2.5 (1.29)*	2.5 (1.29)*
Desyrel	1.5 (0.55)	1.5 (0.55)
Gralise	2.0 (1.09)	1.83 (1.17)
Mobic	3.0 (1.41)†	2.5 (2.12)†
Plavix	2.0 (1.73)*	2.0 (1.73)*
Protonix	2.0 (1.73)‡	1.2 (0.45)‡
Ultram	1.3 (0.57)ŧ	1.0 (0.0)ŧ
Wellbutrin	2.5 (1.56)	2.5 (1.56)
Xanax	2.2 (1.64)**	2.4 (1.52)**
Zoloft	3.2 (2.09)**	1.8 (1.79)**

* No medication guide information for one question and no Google snippet block for one question; total Google snippet blocks analyzed=4;

† Medication guide did not have drug information for four questions; total Google snippet blocks analyzed=2;

‡ No Google snippet block and drug information in the medication guide for one question; total Google snippet blocks analyzed=4;

ŧ Medication guide did not have drug information for three questions; total Google snippet blocks analyzed=3;

** One Google snippet block was not available; total Google snippet blocks analyzed=5

The drug with the highest mean rating for accuracy was Zoloft at 3.2, and the lowest was Ultram with a mean rating of 1.3. Ambien, Mobic, and Wellbutrin had the highest mean rating for completeness of 2.5, and Protonix had the lowest mean rating of 1.0.

For five out of the six questions, the information in the Google snippet blocks had less than 50% accuracy and completeness compared to the medication guides (Table 3).

**Table 3:** Mean accuracy and completeness scores by question

Question	Mean (SD)
**What is [drug] used for?**	
Accuracy	2.70 (1.41)
Completeness	2.10 (1.37)
**How do I take [drug]?** ^*^	
Accuracy	1.00 (0)
Completeness	1.00 (0)
**What are the possible side effects of [drug]?** ^**†**^	
Accuracy	1.75 (1.39)
Completeness	1.00 (0)
**What should I avoid while taking [drug]?** ^**‡**^	
Accuracy	2.00 (1.41)
Completeness	2.20 (1.30)
**What are the ingredients of [drug]?** ^*^	
Accuracy	3.38 (1.51)
Completeness	3.00 (1.41)
**How do I store [drug]?ŧ**	
Accuracy	2.13 (1.35)
Completeness	2.25 (1.49)
**Total**	
Accuracy	2.40
Completeness	1.87

* Did not contain medication guide information for two drugs; total drugs analyzed=8;

† No snippet block available for two drugs; total drugs analyzed=8;

‡ No snippet block or medication guide information available for 5 drugs; total drugs analyzed=5;

ŧ No medication guide information for one drug and no snippet block for two drugs; total drugs analyzed=8

The average accuracy and completeness scores of the Google snippet blocks were highest for the “What are the ingredients of [the drug]?” question, with average scores of 3.38 (51–75%) and 3.00 (51–75%), respectively. The question that had the lowest score was the “How to take [drug]?” with averages of 1.00 (<25%) for both accuracy and completeness.

The sources referenced by the Google snippet blocks included Rxlist.com, which was the most frequently cited by seventeen Google snippet blocks. Everydayhealth.com was cited by seven of the Google snippet blocks; the remaining twenty-two Google snippet blocks were gathered from twelve different websites (Table 4).

**Table 4:** References cited by Google snippets (n=46)

Website	Google snippets
Rxlist	17
EverydayHealth.com	7
WebMD	5
Drugs.com	4
Medlineplus.gov	3
FDA.gov	2
Cardiosmart.com	1
Healthline.com	1
Medicinenet.com	1
Sharecare.com	1
Wikipedia.com	1
Fco.factsandcomparisons.com	1
Nami.org	1
Mayoclinic.com	1

## DISCUSSION

The drug information in Google snippet blocks had less than 50% accuracy and completeness for most of the questions relevant to drug therapy. The Google snippet blocks provided general information and lacked specificity related to certain components of the questions, which contributed to the low accuracy and completeness ratings. Moreover, the majority of the Google snippet blocks did not provide a complete list of common side effects, details about storage conditions, instructions on how to take the drug, or age groups the drug was approved for when compared to the medication guides.

Although some of the questions such as the indications and ingredients may not alter the health of a patient, questions pertaining to the use of a drug may present the most harm. The question “How to take [drug]?” had the lowest ratings for both accuracy and completeness; therefore, individuals relying on Google snippet blocks to provide directions and guidance upon starting a new medication may not take the drug appropriately. Moreover, drugs with a medication guide require proper medication use and adherence to ensure the efficacy of the drug, so it is vital for patients to have accurate and complete instructions on how to take a medication.

An unexpected finding was the inconsistency of drug information provided in the medication guides. For the drugs Ultram, Protonix, Mobic, Plavix, and Ambien, there were several questions that were not included in the medication guide such as “how do I take [drug]?”, “what are the ingredients,” and “what to avoid while on [drug].” Manufacturers are required to follow a specific format and include content in the medication guides according to FDA regulations [12]. A possible explanation for the discrepancy is that these drugs have been on the market for more than ten years, and revisions of the medication guides to the new format may have been overlooked.

This study specifically focused on drug information found in Google snippets; however, the results were consistent with previous studies that evaluated drug information found in open-sourced websites or Google searches when compared to the medication guides [9–10].

There were several limitations in this study. The analysis only consisted of ten drugs from different drug classes, which provided insight into the accuracy and completeness of Google snippet blocks; however, a greater number of drugs may provide more robust data. Also, results may differ with inclusion of drugs administered in the hospital setting. The brand name was used to find the Google snippet blocks for analysis in the study. Some of the branded products may not be available on the market any longer (e.g., Desyrel) or may not be prescribed as often in the clinical setting, which may impact the type and amount of drug information found compared to using the generic name for the search instead. The scoring system was based on other scoring systems used in previous studies; however, it was not validated. Validating the scoring system may provide more reliable and consistent predictive value of the accuracy and completeness ratings. Readability was not evaluated in this study, as information provided by the Google snippet blocks was relatively short and may not have offered an accurate reading level. Future studies may evaluate the length of drug information found in online sources and its impact on consumer and patient preference and understanding of drugs compared to regulated documents such as the medication guides.

Google snippet blocks were designed to provide convenience and assistance with searching information for consumers; however, this study suggests there is inaccurate and incomplete drug information found in the Google snippet blocks when compared to the medication guide. Inaccurate information regarding drugs and health-related information may cause patient harm; therefore, federal stakeholders such as the FDA, National Library of Medicine, or Human Health Services may prevent the potential for harm through policies or guidance on publication of drug information on open-sourced websites. With the lack of regulation currently on the quality and accuracy of drug information, it is imperative for health care and health information professionals to identify reliable resources and refer consumers and patients to these resources if written information is needed about their drugs. In addition, health information professionals and health sciences librarians may collaborate with physicians, pharmacists, and other health care professionals to develop appropriate consumer and patient education materials if reliable resources are not available.

## Data Availability

Data associated with this article are available in the One Drive SharePoint website available at https://kansas-my.sharepoint.com/:f:/g/personal/cnguyen_home_ku_edu/Es3IIS9vpZBOoAdz4G3uOz8B43IPzNnyboCRcNWbVmHJSg?e=fJvyKdurl.
